# Correlation between mean platelet volume and fasting plasma glucose levels in prediabetic and normoglycemic individuals

**DOI:** 10.1186/1475-2840-12-14

**Published:** 2013-01-11

**Authors:** Masanori Shimodaira, Tomohiro Niwa, Koji Nakajima, Mutsuhiro Kobayashi, Norinao Hanyu, Tomohiro Nakayama

**Affiliations:** 1 Department of Internal Medicine, Iida Municipal Hospital, Nagano, Japan; 2 Division of Laboratory Medicine, Department of Pathology and Microbiology, Nihon University School of Medicine, Tokyo, Japan

**Keywords:** Diabetes, Prediabetes, Hyperglycemia, Fasting plasma glucose, Mean platelet volume

## Abstract

**Background:**

Prediabetes is an independent risk factor for cardiovascular diseases. Mean platelet volume (MPV) can reflect platelet activity, and high MPV is associated with thrombogenic activation and an increased risk of cardiovascular disease. In diabetic patients, MPV is higher when compared with normal subjects. However, the relationship between MPV and prediabetes is poorly understood. The purpose of the present study was to compare MPV in prediabetic and normoglycemic subjects, and to evaluate the relationship between MPV and fasting plasma glucose (FPG) levels in these two groups.

**Methods:**

We retrospectively studied 1876 Japanese subjects who had undergone health checks at Iida Municipal Hospital. Age, sex, body mass index (BMI), blood pressure, medical history, smoking habits, alcohol intake, lipid profiles, FPG levels, and MPV were evaluated. Subjects were categorized into four groups according to FPG: Q1 (70 mg/dL ≤ FPG < 90 mg/dL, n = 467), Q2 (90 mg/dL ≤ FPG < 95 mg/dl, n = 457), Q3 (95 mg/dL ≤ FPG < 100 mg/dL, n = 442), and Q4 (100 mg/dL ≤ FPG < 126 mg/dL, n = 512). Q1, Q2, and Q3 were defined as normal FPG groups and Q4 was defined as prediabetic group.

**Results:**

The MPV increased with the increasing FPG levels, in the following order: Q1 (9.89 ± 0.68 fl), Q2 (9.97 ± 0.69 fl), Q3 (10.02 ± 0.72 fl), and Q4 (10.12 ± 0.69 fl). After adjusting for the confounding parameters, MPV of the prediabetic group was higher than that in other groups (P < 0.001 for Q4 vs. Q1 and Q2, and P < 0.05 for Q4 vs. Q3). MPV in the high-normal glucose group (Q3) was significantly higher than in the low-normal glucose group (Q1). MPV was independently and positively associated with FPG, not only in prediabetic subjects but also in normal FPG subjects (β = 0.020 and β = 0.006, respectively).

**Conclusions:**

MPV in patients with prediabetes was higher than that in normal subjects, and was positively associated with FPG levels in prediabetic and normal subjects.

## Background

The criteria for determining prediabetes are generally defined as impaired fasting glucose (IFG) levels, impaired glucose tolerance (IGT), or both [1]. Prediabetes is a preclinical stage in the hyperglycemia continuum where subjects are at increased risk of developing diabetes in the near future [2]. Individuals with prediabetes are at a high risk of not only developing diabetes but also of adverse cardiovascular events (myocardial infarction, stroke, or cardiovascular death) in the later life [3,4].

Platelets play an important role in the normal hemostasis; the mean platelet volume (MPV) and an accurate measure of the platelet size are considered markers and determinants of platelet function. Larger platelets with higher MPV are hemostatically more reactive and produce higher amounts of the prothrombotic factor Thromboxane A2, increasing a propensity to thrombosis [5].

Recently, increased MPV is emerging as an independent risk factor for thromboembolism [6], stroke, and myocardial infarction [7,8]. In patients with diabetes, MPV was higher compared with the normal glycemic controls; in addition, it has been proposed that an increase in MPV may play a role in the micro- and macro-vascular complications related to diabetes [9,10]. Furthermore, platelet activity recovers with improved glycemic control [11]. Although several studies have reported a positive association between MPV and fasting plasma glucose (FPG) levels in diabetes [9,12], few reports have addressed the correlation between MPV and FPG in the general population [13,14]. One article, involving elderly Italian subjects (mean age, 72.9 years), reported a significant positive correlation between MPV and FPG [13]. On the other hand, a second article, involving a Korean population, showed a significant negative correlation between MPV and FPG in normal FPG subjects [14]. These opposing results indicate that the correlation between MPV and FPG in non-diabetic subjects is controversial. Moreover, there are few studies regarding the correlation between MPV and FPG in intermediate hyperglycemic subjects [14].

The aim of the present study was to determine the relationship between MPV and prediabetes and to evaluate the relationship between MPV and FPG levels in a large, non-diabetic Japanese population.

## Methods

### Study population

We investigated 2177 Japanese subjects who visited the health promotion center of Iida Municipal Hospital from October 2011 to September 2012. Seventeen subjects with abnormal platelet counts (< 100 and > 400 × 103/μL) and 53 subjects who were taking antiplatelet medicines (aspirin, ticlopidine, and clopidogrel) were excluded. We excluded 198 diabetic subjects with the history of anti-diabetic therapy, FPG > 125 mg/dL, or HbA1c > 6.4% [15]. Moreover, we excluded three subjects who had extremely low FPG levels (< 70 mg/dL). Three male subjects with hemoglobin levels below 13 mg/dL and 27 female subjects with hemoglobin levels below 12 mg/dL were also excluded from the study because nutritional anemias can cause reactive thrombocytosis and thus increase MPV. The remaining 1876 subjects were included in this study. Informed consent was obtained from all the participating subjects.

The subjects were divided into four groups on the basis of FPG levels. Using the diagnostic criterion of the American Diabetes Association [16], after assigning the IFG subjects to group Q4 (100 mg/dL ≤ FPG < 126 mg/dL, n = 512), FPG levels were categorized into the following tertiles: Q1 (70 mg/dL ≤ FPG < 90 mg/dL, n = 467), Q2 (90 mg/dL ≤ FPG < 95 mg/dL, n = 457), and Q3 (95 mg/dL ≤ FPG < 100 mg/dL, n = 442).

Blood samples were collected in the morning following a fasting period of 12 hours. The venous blood samples were mixed with dipotassium EDTA and tested within 30 minutes of collection to minimize variations due to sample aging. MPV and platelets were measured using an automatic blood counter (XE-5000, Sysmex Corp., Japan). Glucose, uric acid, and lipid profiles were determined by standard methods.

Following were the criteria for dyslipidemia: serum LDL-cholesterol ≥ 140 mg/dL, HDL-cholesterol < 40 mg/dL, triglycerides ≥ 150 mg/dL, or having been treated for dyslipidemia. Hypertension was defined as systolic blood pressure ≥ 140 mmHg, diastolic blood pressure ≥ 90 mmHg, or presently taking any medication prescribed for hypertension. BMI was calculated as weight (kg)/height (m^2^).

A questionnaire was used to obtain information about familial medical history and the subjects’ lifestyle, such as smoking habits and alcohol ingestion. Familial history of diabetes was defined as having one or more relatives (parent or sibling) with diabetes. Individuals who had smoked < 100 cigarettes during their lifetime were considered nonsmokers, those who had smoked ≥ 100 cigarettes and were currently not smoking were considered former smokers, and those who had smoked ≥ 100 cigarettes and were currently smoking were considered current smokers. The following criteria were defined for alcohol consumption groups: drinking never or rarely (0–5 times/year), occasionally (1–5 times/month), and regularly (1–7 times/week).

### Statistical analysis

Statistical analyses were performed using the SPSS software version 15.0 (SPSS Inc. IL, USA). The clinical characteristics of the four groups were compared using one-way analysis of variance (ANOVA) for continuous variables, whereas the chi-squared test was used to compare the categorical parameters.

Pearson’s correlation coefficients were calculated to evaluate the relationships between MPV and several clinical variables [age, sex, blood pressure (systolic/diastolic), BMI, uric acid, total cholesterol, HDL-cholesterol, LDL-cholesterol, smoking, and alcohol ingestion]. The distribution of triglycerides was skewed; hence, we conducted Pearson’s linear correlation using log-transformed values instead of the raw data. To assess independent relationships between MPV and the clinical variables, a multiple linear regression analysis was performed. MPV of the four groups were analyzed using ANCOVA test, considering confounding factors. Data were expressed as mean ± standard deviation. P values less than 0.05 were considered statistically significant.

## Results

The characteristics of the 1876 subjects enrolled in this study are displayed in Table 1. The MPV increased with the increasing FPG levels. In addition, age, male/female ratio, BMI, systolic blood pressure, diastolic blood pressure, uric acid, lipid profiles, smoking status, and alcohol ingestion differed in the four groups.

**Table 1:** Clinical and metabolic characteristics of study participants according to fasting plasma glucose

	**Q1**	**Q2**	**Q3**	**Q4**	**P-values**
	**(n=467)**	**(n=455)**	**(n=442)**	**(n=512)**	
FPG (mg/dl)	84.63±3.83	92.05±1.41	96.73±1.45	106.17±5.79	
MPV (fl)	9.89±0.68	9.97±0.69	10.02±0.72	10.12±0.69	<0.001
Count of platlets (104/μL)	21.38±5.80	21.61±4.46	21.37±4.81	21.38±4.96	0.869
Men (%)	38.8%	56.70%	63.1%	68.8%	<0.001
Age (years)	51.40±11.52	51.99±11.08	54.16±10.88	57.41±10.32	<0.001
BMI (kg/m2)	21.48±2.82	22.31±3.03	22.89±3.18	23.67±3.28	<0.001
SBP (mmHg)	113.33±15.70	117.87±15.96	117.79±15.28	124.24±14.66	<0.001
DBP (mmHg)	66.78±11.12	70.35±11.05	70.12±11.00	74.73±10.32	<0.001
UA (mg/dl)	4.95±1.21	5.40±1.36	5.51±1.31	5.75±1.33	<0.001
TC (mg/dl)	203.80±34.36	206.27±33.28	206.83±32.77	210.20±33.62	0.028
TG (mg/dl)	89.63±68.38	107.26±92.24	109.92±68.34	122.91±80.18	<0.001
HDL-C (mg/dl)	66.94±15.61	63.20±14.15	60.79±14.23	60.73±15.02	<0.001
LDL-C (mg/dl)	114.65±31.15	118.67±30.40	121.37±29.88	122.70±28.78	<0.001
Hypertension (%)	12.0%	15.60%	20.6%	30.1%	<0.001
Dyslipidemia (%)	34.7%	48.13%	51.8%	59.6%	<0.001
Familial hisory of diabetes (%)	13.9%	13.63%	15.6%	16.4%	<0.001
Smoking status (%)					
current	16.7%	22.64%	18.8%	17.4%	<0.001
former	21.8%	24.40%	31.0%	36.1%	
never	61.5%	52.97%	50.2%	46.5%	
Alcohol ingestion (%)					
regularly	21.0%	26.59%	29.0%	41.2%	<0.001
occasionally	39.4%	37.58%	36.7%	28.9%	
never or rarely	39.6%	35.82%	34.4%	29.9%	

Data are shown as the mean ± standard deviation and percentage (%).

P values were calculated using the ANOVA and X2-tests.

FPG, fasting plasma glucose; MPV, mean platelet volume; BMI, body mass index.

SBP, systolic blood pressure; DBP, diastolic blood pressure.

UA, uric acid; TC, total cholesterol; TG, triglyceride; LDL-C, LDL-cholesterol; HDL-C, HDL-cholesterol.

In all the groups (Q1–Q4), a significant correlation was observed between MPV and FPG, sex, age, BMI, systolic blood pressure, diastolic blood pressure, and uric acid (Table 2). In addition, not only in the prediabetic group (Q4) but also in normal FPG groups (Q1–Q3), MPV correlated with FPG. However, lipid profiles, smoking habits, and alcohol ingestion were not correlated with MPV. After adjusting for these confounding factors using multiple linear regression analysis, MPV was found to be independently and positively associated with FPG, not only in prediabetic subjects but also in the subjects with normal FPG levels (Table 3). This correlation was stronger in prediabetic subjects than in normal FPG subjects (β = 0.020 and β = 0.006, respectively).

**Table 2:** Correlations between mean platelet volumes and various parameters

	**MPV in total subjects (Q1~Q4)**		**MPV in normal FPG subjects (Q1~Q3)**		**MPV in prediabetic subjects (Q4)**	
	**n=1876**		**n=1364**		**n=512**	
	**r**	**P-values**	**r**	**P-values**	**r**	**P-values**
FPG (mg/dl)	0.25	P<0.001	0.17	P<0.01	0.28	P<0.001
Sex	0.1064	P<0.001	0.1064	P<0.001	0.1064	P<0.001
Age (years)	0.08	P<0.001	0.07	P<0.05	0.03	NS
BMI (kg/m2)	0.06	P<0.05	0.04	NS	0	NS
SBP (mmHg)	0.09	P<0.001	0.09	P<0.001	0.08	NS
DBP (mmHg)	0.1	P<0.001	0.07	P<0.05	0.07	NS
Uremic Acid (mg/dl)	0.1	P<0.001	0.09	P<0.001	0.08	NS
TC (mg/dl)	-0.01	NS	0.01	NS	-0.01	NS
Log TG	0.01	NS	0.03	NS	0.03	NS
HDL-C (mg/dl)	-0.02	NS	-0.02	NS	-0.01	NS
LDL-C (mg/dl)	-0.01	NS	0.0041	NS	0.0934	NS
Hypertension	0.0586	P<0.05	0.0263	NS	0.0782	NS
Dyslipidemia	0.0121	NS	0.0040	NS	0.0782	NS
Familial hisory of diabetes	0.0424	NS	0.0229	NS	0.0106	NS
Smoking status	0.0233	NS	0.0233	NS	0.0233	NS
Alcohol ingestion	0.0026	NS	0.0004	NS	0.0012	NS

Coefficients (r) and P-values are calculated using the Pearson’s correlation model.

FPG, fasting plasma glucose; MPV, mean platelet volume; BMI, body mass index; SBP, systolic blood pressure; DBP, diastolic blood pressure.

UA, uremic acid; TC, total cholesterol; Log TG, log-transformed triglyceride; LDL-C, LDL-cholesterol; HDL-C, HDL-cholesterol; NS, not significant.

**Table 3:** Multiple linear regression analyses conducted to assess independent relationships between MPV and clinical variables

	**MPV in total subjects (Q1~Q4)**		**MPV in normal FPG subjects (Q1~Q3)**		**MPV in prediabetic subjects (Q4)**	
	**n=1876**		**n=1364**		**n=512**	
	**β**	**P-values**	**β**	**P-values**	**β**	**P-values**
FPG (mg/dl)	0.009	P<0.001	0.006	P<0.05	0.020	P<0.001
Sex	0.078	NS	0.073	NS	0.114	NS
Age (years)	0.002	P<0.05	0.003	NS	0.001	NS
BMI (kg/m2)	0.004	NS	0.003	NS	0.006	NS
SBP (mmHg)	0.001	NS	0.001	NS	0.002	NS
DBP (mmHg)	0.002	NS	0.002	NS	0.001	NS
UA (mg/dl)	0.021	NS	0.006	NS	0.014	NS
	R2=0.33, adjusted R2= 0.30, P<0.001		R2=0.19, adjusted R2= 0.14, P<0.001		R2=0.43, adjusted R2= 0.30, P<0.01	

FPG, fasting plasma glucose; MPV, mean platelet volume; BMI, body mass index.

SBP, systolic blood pressure; DBP, diastolic blood pressure; UA, uric acid; NS, not significant.

**Figure 1:**
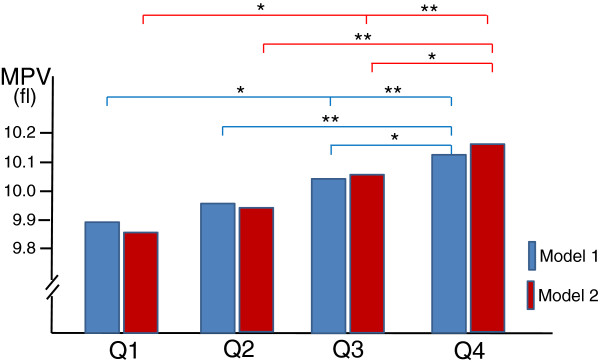
**MPV in different fasting plasma glucose groups in noncdiabetic subjects.** One asterisk means P < 0.05, two asterisks mean P < 0.01. Model 1; adjusted for age and sex. Model 2; adjusted for age, sex, systolic blood pressure, diastolic blood pressure, BMI, uremic acid, total cholesterol, triglyceride, HDL-cholesterol, LDL-cholesterol, smoking, and alcohol ingestion.

As shown in Figure 1, even after adjusting for the confounding parameters, MPV in the prediabetic group (Q4) was higher than in other groups. Moreover, MPV of the high-normal glucose group (Q3) was higher than that of the low-normal glucose group (Q1) even after adjustments of the parameters.

## Discussion

MPV is modified by various biosocial and lifestyle factors such as race, gender, age, blood pressure, smoking habits, and alcohol consumption [17,18]. In a previous study, MPV could be positively correlated with fasting glucose levels in diabetic and prediabetic groups; however, the sample size was modest (50 diabetic subjects and 50 prediabetic subjects) and some confounding factors were not investigated sufficiently [19]. Muscari et al. [13] showed that MPV values were associated with FPG in Italian subjects. However, these subjects were not entirely representative of the general population because most of the subjects were elderly (mean age, 72.9 years), hypertensive (86%), hypercholesterolemic (47%), and overweight or obese (46%).

In the present study, we investigated the relation of MPV and FPG levels in general population using an adequate sample size, and observed that MPV in prediabetic subjects was higher than that in normoglycemic subjects. Furthermore, MPV of the high-normal glucose subjects was higher than that of the low-normal glucose subjects. Moreover, we observed a positive correlation between MPV and FPG levels, not only in the prediabetic but also in the normoglycemic subjects, independent from variable factors. We excluded subjects with extremely low FPG levels (< 70 mg/dL); therefore, there is a possibility that MPV follows a J-shaped curve at the low glucose levels. However, at least in the physiological glycemic range, the correlation between MPV and FPG levels was confirmed. Unlike our results, Kim et al. [14] reported a negative correlation between MPV and FPG in Korean subjects with normal glucose tolerance and intermittent hyperglycemia, although intermediate hyperglycemia is associated with an increased risk of cardiovascular diseases. In contrast, we evaluated triglyceride and uric acid levels and alcohol consumption as confounding factors and reported that MPV can differ based on individual characteristics, including lipid profiles, alcohol intake, genetics, race/ethnicity, and different populations.

The definition of IFG is not consistent worldwide. According to the American Diabetes Association (ADA) criterion, IFG is defined as FPG levels of 100–125 mg/dL; this threshold was lowered in 2003 for better prediction of future diabete incidence [20]. Other organizations, including the European Diabetes Epidemiology Group (EDEG) and the Japan Diabetes Society (JDS), have retained the original diagnostic range for IFG at FPG levels of 110–125 mg/dL [21,22]. In the present study, we adopted ADA criterion, more strictly than EDEG and JDS criteria, in order to distinguish prediabetic subjects from normoglycemic subjects more efficiently. These stringent criteria ensured increased sample confidence in our study.

The term “prediabetes” has replaced the clinical definitions known as borderline or chemical diabetes, traditionally used to identify the individuals at high risk of progression to overt diabetes. Prediabetes has been linked to a modest increase in overall cardiovascular events and has been associated with a higher risk of stroke [4,23]. Moreover, it has been reported that in prediabetic individuals, the von Willebrand factor levels, essential for platelet aggregation and adhesion, is significantly higher than in the controls, and Willebrand factor levels were positively correlated with MPV in the prediabetic group (r = 0.452, P = 0.001) [24]. In our study, MPV in the prediabetic subjects (Q4) was significantly higher than those in low-normal glucose group (Q1), middle-normal glucose group (Q2), and high-normal glucose group (Q3). Our results suggest that the subjects with prediabetes tend to have increased MPV that could have contributed to an increased risk of cardiovascular disease.

A study using a large multiethnic cohort has demonstrated that the risk of cardiovascular events or death in normoglycemic and prediabetic subjects increases progressively with increasing FPG levels. A 1 mmol/l (18 mg/dl) increase in FPG has been associated with a 17% increase in the risk of future cardiovascular events or death [25]. Even within the normoglycemic range, elevated cardiovascular risk is strongly and independently associated with glucose levels. Subjects with fasting glucose levels in the high-normal range (95–99 mg/dL) have an increased cardiovascular risk when compared with subjects in low-normal range (< 80 mg/dL) [26]. In the present study, MPV in the high-normal glucose group (Q3) was higher than that in the low-normal glucose group (Q1). Although the underlying mechanism of higher MPV in Q3 subjects remains unclear, it has been suggested that increased MPV may be due to osmotic swelling as a result of hyperglycemia [27]. Another postulated mechanism from a study in mice demonstrated that insulin induces megakaryocytes to produce larger platelets [28].

Obesity is a risk factor of cardiovascular disorders, partly due to increased oxidative stress and inflammation, which are associated with increased reactive oxygen species (ROS) production and decreased NO bioavailability. Recently, Monteiro et al. [29] showed that metabolic abnormalities, as a consequence of high-fat diets, cause platelet hyperaggregability involving enhanced intraplatelet ROS production and decreased NO bioavailability. In mildly hypertriglyceridemic subjects, n-3 polyunsaturated fatty acids increased MPV values slightly [30]. Although we did not measure dietary fat intake in our subjects, there is a possibility that a high-fat diet increases MPV. In obese subjects, MPV was positively correlated with BMI and a positive correlation was also shown between weight loss and reduction in MPV [31]. A higher BMI value was strongly associated with higher insulin levels and insulin resistance. In subjects with cardiovascular disease, MPV was significantly elevated in those with insulin resistance when compared to insulin-sensitive subjects [32]. However, there are few reports regarding the correlation between MPV and insulin level in the general population. Nonetheless, MPV was positively associated with insulin level in polycystic ovary syndrome, which is related to increased insulin levels and the incidence of obesity [33]. Therefore, hyperinsulinemia that accompanies obesity may influence platelet reactivity in obese patients.

There are several limitations to our study. It has been shown that up to five percent of subjects with IFG appear to have diabetes as per the results of the 2-hour glucose tolerance tests [34,35]. True diabetic and prediabetic groups are demarcated by glucose tolerance tests. Therefore, some of the people in the prediabetic group might have had diabetes. Second, Our study was retrospective and we did not determine the relationship between MPV and the clinical events. Lastly, our present study only included Japanese subjects, in which the prevalence of obesity (BMI > 30) was < 3%, in contrast to > 30% in Europeans and Americans [36]. Therefore, a duplicate study with other populations is indispensable to confirm our results.

Using a representative sample of Japanese adults, we found that the prediabetic subjects had higher MPV than the control individuals. Furthermore, MPV could be positively and independently correlated with the FPG levels, not only in the prediabetic subjects but also in normoglycemic subjects, after correcting for confounding variables.

## Conclusions

We demonstrated that MPV in prediabetic subjects was higher than that in controls. Moreover, even within the normal range, subjects with high-normal FPG levels had higher MPV than the individuals with low FPG levels. In prediabetic and normoglycemic subjects, MPV showed positive correlation with FPG levels.

## Competing interests

The authors declare that they have no competing interests.

## Authors’ contributions

MS participated in the design of the study, statistical analysis, and preparation of the manuscript. TN and NH contributed to the enrollment of the subjects. KM, MK, and TN critically revised the manuscript for important intellectual content, and all authors have given their final approval.

## References

[B1] American Diabetes Association Diagnosis and classification of diabetes mellitus Diabetes Care 2011 34 1 S62 S69 21193628 10.2337/dc11-S062 PMC3006051

[B2] American Diabetes Association Summary of revisions to the 2011 clinical practice recommendations Diabetes Care 2011 34 1 S3 21193626 10.2337/dc11-S003 PMC3006057

[B3] DeFronzo RA Abdul-Ghani M Assessment and treatment of cardiovascular risk in prediabetes: impaired glucose tolerance and impaired fasting glucose Am J Cardiol 2011 108 3B 24B 10.1016/j.amjcard.2011.03.013 21802577

[B4] Lee M Saver JL Hong KS Song S Chang KH Ovbiagele B Effect of pre-diabetes on future risk of stroke: meta-analysis BMJ 2012 344 e3564 10.1136/bmj.e3564 22677795 PMC3370083

[B5] Vizioli L Muscari S Muscari A The relationship of mean platelet volume with the risk and prognosis of cardiovascular diseases Int J Clin Pract 2009 63 1509 1515 10.1111/j.1742-1241.2009.02070.x 19769707

[B6] Park Y Schoene N Harris W Mean platelet volume as an indicator of platelet activation: methodological issues Platelets 2002 13 301 306 10.1080/095371002220148332 12189016

[B7] Pizzulli L Yang A Martin JF Luderitz B Changes in platelet size and count in unstable angina compared to stable angina or non-cardiac chest pain Eur Heart J 1998 19 80 84 10.1053/euhj.1997.0747 9503179

[B8] Tavil Y Sen N Yazici HU Hizal F Abaci A Cengel A Mean platelet volume in patients with metabolic syndrome and its relationship with coronary artery disease Thromb Res 2007 120 245 250 10.1016/j.thromres.2006.10.005 17145074

[B9] Papanas N Symeonidis G Maltezos E Mavridis G Karavageli E Vosnakidis T Lakasas G Mean platelet volume in patients with type 2 diabetes mellitus Platelets 2004 15 475 478 10.1080/0953710042000267707 15763888

[B10] Hekimsoy Z Payzin B Ornek T Kandogan G Mean platelet volume in type 2 diabetic patients J Diabetes Complications 2004 18 173 176 10.1016/S1056-8727(02)00282-9 15145330

[B11] Demirtunc R Duman D Basar M Bilgi M Teomete M Garip T The relationship between glycemic control and platelet activity in type 2 diabetes mellitus J Diabetes Complications 2009 23 89 94 10.1016/j.jdiacomp.2008.01.006 18358749

[B12] Shah B Sha D Xie D Mohler ER 3rd Berger JS The relationship between diabetes, metabolic syndrome, and platelet activity as measured by mean platelet volume: the national health and nutrition examination survey, 1999–2004 Diabetes Care 2012 35 1074 1078 10.2337/dc11-1724 22410814 PMC3329806

[B13] Muscari A De Pascalis S Cenni A Ludovico C Castaldini N Antonelli S Bianchi G Magalotti D Zoli M Determinants of mean platelet volume (MPV) in an elderly population: relevance of body fat, blood glucose and ischaemic electrocardiographic changes Thromb Haemost 2008 99 1079 1084 18521511 10.1160/TH07-12-0712

[B14] Kim JH Kang SB Kang JI Kim JW Kim SY Bae HY The relationship between mean platelet volume and fasting plasma glucose differs with glucose tolerance status in a korean general population: gender differences Platelets 2012 in press 10.3109/09537104.2012.715214 22916798

[B15] Genuth S Alberti KG Bennett P Buse J Defronzo R Kahn R Kitzmiller J Knowler WC Lebovitz H Lernmark A Follow-up report on the diagnosis of diabetes mellitus Diabetes Care 2003 26 3160 3167 14578255 10.2337/diacare.26.11.3160

[B16] American Diabetes Association Diagnosis and classification of diabetes mellitus Diabetes Care 2008 31 1 S55 60 18165338 10.2337/dc08-S055

[B17] Sundaram M Mohanakrishnan J Murugavel KG Shankar EM Solomon S Srinivas CN Solomon SS Pulimi S Piwowar-Manning E Dawson S Ethnic variation in certain hematological and biochemical reference intervals in a south Indian healthy adult population Eur J Intern Med 2008 19 46 50 10.1016/j.ejim.2007.06.010 18206601

[B18] Cao X Xie X Zhou J Yang P Wang Y Chen Z Increased platelet volume in a general population with prehypertension: a cross-sectional study of 80 545 participants from china Hypertens Res 2012 35 903 908 10.1038/hr.2012.62 22573201

[B19] Coban E Bostan F Ozdogan M The mean platelet volume in subjects with impaired fasting glucose Platelets 2006 17 67 69 10.1080/09537100500220729 16308190

[B20] Expert Committee on the Diagnosis and Classification of Diabetes Mellitus Report of the expert committee on the diagnosis and classification of diabetes mellitus Diabetes Care 2003 26 1 S5 20 12502614 10.2337/diacare.26.2007.s5

[B21] Kuzuya T Nakagawa S Satoh J Kanazawa Y Iwamoto Y Kobayashi M Nanjo K Sasaki A Seino Y Ito C Report of the committee on the classification and diagnostic criteria of diabetes mellitus Diabetes Res Clin Pract 2002 55 65 85 10.1016/S0168-8227(01)00365-5 11755481

[B22] Forouhi NG Balkau B Borch-Johnsen K Dekker J Glumer C Qiao Q Spijkerman A Stolk R Tabac A Wareham NJ The threshold for diagnosing impaired fasting glucose: a position statement by the european diabetes epidemiology group Diabetologia 2006 49 822 827 16525842 10.1007/s00125-006-0189-4

[B23] Ford ES Zhao G Li C Pre-diabetes and the risk for cardiovascular disease: a systematic review of the evidence J Am Coll Cardiol 2010 55 1310 1317 10.1016/j.jacc.2009.10.060 20338491

[B24] Bostan F Coban E The relationship between levels of von willebrand factor and mean platelet volume in subjects with isolated impaired fasting glucose Med Sci Monit 2011 17 PR1 4 21629201 10.12659/MSM.881786 PMC3539533

[B25] Anand SS Dagenais GR Mohan V Diaz R Probstfield J Freeman R Shaw J Lanas F Avezum A Budaj A Glucose levels are associated with cardiovascular disease and death in an international cohort of normal glycaemic and dysglycaemic men and women: the EpiDREAM cohort study Eur J Prev Cardiol 2012 19 755 764 10.1177/1741826711409327 21551215

[B26] Shaye K Amir T Shlomo S Yechezkel S Fasting glucose levels within the high normal range predict cardiovascular outcome Am Heart J 2012 164 111 116 10.1016/j.ahj.2012.03.023 22795290 PMC4934381

[B27] Martyn CN Matthews DM Popp-Snijders C Tucker J Ewing DJ Clarke BF Effects of sorbinil treatment on erythrocytes and platelets of persons with diabetes Diabetes Care 1986 9 36 39 10.2337/diacare.9.1.36 3948646

[B28] Watanabe Y Kawada M Kobayashi B Effect of insulin on murine megakaryocytopoiesis in a liquid culture system Cell Struct Funct 1987 12 311 316 10.1247/csf.12.311 3304669

[B29] Huang Y Yang Z Ye Z Li Q Wen J Tao X Chen L He M Wang X Lu B Lipocalin-2, glucose metabolism and chronic low-grade systemic inflammation in Chinese people Cardiovasc Diabetol 2012 11 11 10.1186/1475-2840-11-11 22292925 PMC3295671

[B30] Park Y Harris WS Dose-dependent effects of n-3 polyunsaturated fatty acids on platelet activation in mildly hypertriglyceridemic subjects J Med Food 2009 12 809 813 10.1089/jmf.2008.1097 19735181

[B31] Coban E Yilmaz A Sari R The effect of weight loss on the mean platelet volume in obese patients Platelets 2007 18 212 216 10.1080/09537100600975362 17497433

[B32] Varol E Akcay S Ozaydin M Erdogan D Dogan A Altinbas A Mean platelet volume is associated with insulin resistance in non-obese, non-diabetic patients with coronary artery disease J Cardiol 2010 56 154 158 10.1016/j.jjcc.2010.03.005 20430587

[B33] Kebapcilar L Taner CE Kebapcilar AG Sari I High mean platelet volume, low-grade systemic coagulation and fibrinolytic activation are associated with androgen and insulin levels in polycystic ovary syndrome Arch Gynecol Obstet 2009 280 187 193 10.1007/s00404-008-0884-0 19107500

[B34] Rerkpattanapipat P D'Agostino RB Jr Link KM Shahar E Lima JA Bluemke DA Sinha S Herrington DM Hundley WG Location of arterial stiffening differs in those with impaired fasting glucose versus diabetes: implications for left ventricular hypertrophy from the multi-ethnic study of atherosclerosis Diabetes 2009 58 946 953 10.2337/db08-1192 19136657 PMC2661581

[B35] Unwin N Shaw J Zimmet P Alberti KG Impaired glucose tolerance and impaired fasting glycaemia: the current status on definition and intervention Diabet Med 2002 19 708 723 12207806 10.1046/j.1464-5491.2002.00835.x

[B36] Examination Committee of Criteria for 'Obesity Disease' in Japan, Japan Society for the Study of Obesity New criteria for 'obesity disease' in Japan Circ J 2002 66 987 992 10.1253/circj.66.987 12419927

